# Lemon Balm and Its Constituent, Rosmarinic Acid, Alleviate Liver Damage in an Animal Model of Nonalcoholic Steatohepatitis

**DOI:** 10.3390/nu12041166

**Published:** 2020-04-22

**Authors:** Myungsuk Kim, GyHye Yoo, Ahmad Randy, Yang-Ju Son, Chi Rac Hong, Sang Min Kim, Chu Won Nho

**Affiliations:** 1 Smart Farm Research Center, Korea Institute of Science and Technology, Gangneung, Gangwon-do 25451, Korea; g-sstainer@kist.re.kr (M.K.); loshell@kist.re.kr (G.Y.); yangjuson@kist.re.kr (Y.-J.S.); crhong@kist.re.kr (C.R.H.); kimsm@kist.re.kr (S.M.K.); 2 Research Center for Chemistry, Indonesian Institute of Sciences (LIPI), Kawasan Puspiptek, Serpong 15314, Indonesia; ahmad.randy@lipi.go.id

**Keywords:** nonalcoholic steatohepatitis (NASH), lemon balm extract (LBE), rosmarinic acid (RA), AMP-activated protein kinase (AMPK), antioxidants, anti-inflammation

## Abstract

Nonalcoholic fatty liver disease (NAFLD) ranges in severity from hepatic steatosis to cirrhosis. Lemon balm and its major constituent, rosmarinic acid (RA), effectively improve the liver injury and obesity; however, their therapeutic effects on nonalcoholic steatohepatitis (NASH) are unknown. In this study, we investigated the effects of RA and a lemon balm extract (LBE) on NAFLD and liver fibrosis and elucidated their mechanisms. Palmitic acid (PA)-exposed HepG2 cells and db/db mice fed a methionine- and choline-deficient (MCD) diet were utilized to exhibit symptoms of human NASH. LBE and RA treatments alleviated the oxidative stress by increasing antioxidant enzymes and modulated lipid metabolism-related gene expression by the activation of adenosine monophosphate-activated protein kinase (AMPK) in vitro and in vivo. LBE and RA treatments inhibited the expression of genes involved in hepatic fibrosis and inflammation in vitro and in vivo. Together, LBE and RA could improve liver damage by non-alcoholic lipid accumulation and may be promising medications to treat NASH.

## 1. Introduction

Nonalcoholic steatohepatitis (NASH), one of the nonalcoholic fatty liver diseases (NAFLDs), accompanied by asymptomatic hepatic steatosis and fibrosis [1]. Although the precise mechanism underlying the progression from fatty liver to NASH has not been determined, the pathogenesis of NASH is believed to involve a multi-hit model; hepatic lipid accumulation, and inflammatory responses mediated by oxidative stress [2,3]. Dietary habits and the imbalance between energy intake and expenditure result in accumulation of lipids in the liver, which is a first hit leading to NASH. Excess hepatic lipids increase free fatty acids, which are converted to lipotoxic lipids, leading to the production of reactive oxygen species as well as inflammatory responses. These processes consequently result in NASH. The ideal treatment for NASH should not only reverse the accumulation of triglycerides (TGs) in hepatocytes but also effectively suppress hepatic inflammations [4]. No pharmacological agent has been approved by the U.S. Food and Drug Administration for the treatment of NAFLD/NASH, and therefore, there is an urgent need for the development of effective treatments.

Adenosine monophosphate-activated protein kinase (AMPK) is a serine/threonine kinase, and the AMPK pathway plays a pivotal role in regulating the energy balance in cells [5]. Under low adenosine triphosphate (ATP) -to- adenosine monophosphate (AMP) ratio, AMPK is activated by phosphorylation of its α-subunit and directs the inhibition of anabolic pathways, accompanied by the induction of catabolic pathways. AMPK activation suppresses fatty acid synthesis, enhances β-oxidation, and induces anti-inflammation by decreasing the activation of hepatic stellate cells [5,6]. In an animal model of NASH, AMPK activation decreased hepatic lipid accumulation and improved insulin resistance. Furthermore, recent reports have suggested that nuclear factor erythroid 2-related factor 2 (NRF2), a major regulator of the redox status, is a potential target of AMPK, and AMPK inhibits the inflammation [7,8]. Considering the two-hit hypothesis of NASH pathogenesis, involving lipid accumulation and inflammation with oxidative stress, the activation of AMPK is regarded as one of potential targets for NAFLD/NASH.

Lemon balm (*Melissa officinalis*) has been used as a medicinal plant to treat anxiety, gastrointestinal disorders, and Alzheimer’s disease [9,10,11]. Moreover, it has an antioxidant activity and protects against oxidative stress-induced apoptosis [12]. An ethyl acetate fraction of lemon balm extract has been shown to reduce high-fat-induced NAFLD in mice [13,14]. The main active compound of lemon balm is rosmarinic acid (RA, *O*-caffeoyl-3,4-dihydroxyphenyl lactic acid), which possesses a multitude of biological activities; the antioxidant, anti-inflammatory, anti-fatty liver, and anti-obesity effects [15,16,17]. However, it remains unknown whether lemon balm extract or RA exerts beneficial effects on NASH, and their molecular mechanisms of action have not been elucidated. In this study, we evaluated whether lemon balm extract and RA could suppress the pathogenesis of NASH.

## 2. Materials and Methods

### 2.1. Plant Material and Preparation of RA

Aerial parts of lemon balm were supplied by Richwood Pharma Co., Ltd. (Seoul, Korea). One kilogram of dried aerial parts of lemon balm was extracted by refluxing EtOH (0–100%) in de-ionized water for 4 h and then filtered through Whatman No. 1 filter paper (GE Healthcare Life Sciences, Pittsburgh, PA, USA). RA was identified from lemon balm (3.59% in lemon balm extract, LBE) as described previously [18].

### 2.2. Reagents and Materials

Dulbecco’s modified Eagle’s medium (DMEM) and fetal bovine serum (FBS) were purchased from HyClone Laboratories (Logan, UT, USA). Palmitic acid (PA), 3-(4,5-dimethylthiazol-2-yl)-2,5-diphenyltetrazolium bromide (MTT), Oil Red O, and other reagents were purchased from Sigma Chemical Co. (St. Louis, MO, USA). Primary antibodies were purchased from Santa Cruz Biotechnology (Santa Cruz, CA, USA) and Cell Signaling Technologies (Beverly, MA, USA) (Supplementary Table S1).

### 2.3. Cell Culture

HepG2 hepatocellular carcinoma cells (American Type Culture Collection, Manassas, VA, USA) were grown in DMEM supplemented with 10% FBS and 100 U/mL penicillin at 37 °C in an atmosphere of 5% CO_2_. For experiments, cells were incubated in the presence or absence of PA, which induces lipid accumulation in hepatocytes, for 24 h and then in the presence or absence of LBE (50 or 100 μg/mL) or RA (20 or 40 μM) for 24 h.

### 2.4. Chemical Analysis

The concentration of RA in lemon balm extracts was determined using an Agilent 1260 Infinity Liquid Chromatograph (Agilent Technologies, Santa Clara, CA, USA), equipped with a quaternary pump delivery system (G1331B), auto-sampler (G1329B), column thermostat (G1316A), and diode array and multiple wavelength detector (G1315D). After pretreatment, an amount equivalent to 10 μL of each sample was injected into a Capcell Pak C18-Column (5 μm, 4.6 × 250 mm, Shiseido, Tokyo, Japan) at 40 °C, with detection at λ = 330 nm. The mobile phase A was 0.1% (*v*/*v*) formic acid in water, while mobile phase B was acetonitrile. The separation was obtained at a flow rate of 0.8 mL/min with a gradient program that allowed for 5 min at 10% B followed by a 35 min step that raised eluent B to 100%, after then holding 5 min. Total analysis time was 45 min.

### 2.5. Animals and Treatments

Of thirty-five four-week-old male db/db mice (Central Laboratory Animal, Inc., Seoul, Korea), 28 were fed the methionine- and choline-deficient (MCD, Central Laboratory Animal, Inc.) diet, and seven were fed a regular chow (control group) for 2 weeks. The 28 mice were divided into four groups (*n* = 7 each) and treated with LBE (200 mg/kg daily), RA (10 or 30 mg/kg daily), or the vehicle alone (MCD group) by oral gavage for an additional 2 weeks while continuing to be fed the MCD diet. The control and MCD diet-fed mice were administered an equal volume of the vehicle (carboxymethyl cellulose). The body weight and food intake were measured twice weekly.

At the end of the treatment period, all mice were fasted overnight and sacrificed by intraperitoneal injection of a Zoletil–Rompun mixture. The livers and the blood were collected and stored at −80 °C. The experimental protocol was approved by the Animal Use and Care Committee of the Korea Institute of Science and Technology (2015-012; Seoul, Korea).

### 2.6. Histopathological Analysis

Liver tissues were fixed in 10% formalin, embedded in paraffin, sectioned, and stained with Hematoxylin and eosin (H&E). Frozen livers embedded at optimal cutting temperature were sectioned at a thickness of 4 μm using a cryostat, fixed in 4% (*v*/*v*) formalin for 10 min, and stained with an Oil Red O working solution.

### 2.7. Biochemical Analysis

The activities of alanine transaminase (ALT) and aspartate transaminase (AST) in the serum were determined using ALT and AST activity assay kits, respectively (BioVision, Minneapolis, MN, USA). Hepatic TG levels were measured using TG kit (Cayman Chemical, Ann Arbor, MI, USA).

### 2.8. Determination of the Liver Collagen Content

Paraffin-embedded liver sections were stained for collagen with Sirius Red, as described previously [19]. Hepatic hydroxyproline was measured using a hydroxyproline colorimetric assay kit (BioVision) to quantify the liver collagen content.

### 2.9. Western Blot Analysis

Homogenized liver specimens (approximately 100 mg each) or HepG2 cells were lysed in lysis buffer supplemented with a protease inhibitor cocktail (Sigma–Aldrich). Protein concentrations were measured by the Bradford assay. Western blot analysis was performed according to a standard procedure using the specific antibodies (Supplementary Information Table S1). Briefly, protein samples were resolved on a 10% sodium dodecyl sulfate-polyacrylamide gel. These proteins were then transferred to polyvinylidene difluoride membranes. The membranes were blocked with PBS-T containing 3% bovine serum albumin (BSA). Subsequently, the membranes were reacted with specific antibodies at 1/1000 and subsequently specific secondary antibodies at 1/3000. Proteins were detected using an enhanced chemiluminescence detection system (Amersham, Buckinghamshire, UK) and visualized using a LAS-3000 luminoimager (Fuji Film Co., Tokyo, Japan). The density of each band relative to that of the β-actin band was determined using the luminoimager (*n* = 3).

### 2.10. Quantitative Real-Time Reverse Transcription–Polymerase Chain Reaction

Total ribonucleic acid (RNA) was isolated from liver specimens and HepG2 cells using the TRIzol reagent (Invitrogen, Carlsbad, CA, USA), and the RNA concentration was quantified spectrophotometrically at 260 nm. cDNA was synthesized from 2 µg of total RNA in a reaction mixture containing oligo (dT) and a reverse transcription premix (ELPIS-Biotech, Daejeon, Korea). Quantitative real-time PCR (qPCR) was performed using a SYBR Green master mix (Roche, Basel, Switzerland) in a Light Cycler 480 real-time PCR system (Roche) and the following conditions: 40 cycles of 95 °C for 30 s, 55 °C for 30 s, and 72 °C for 30 s. mRNA expression levels of mouse genes were determined using the specific primers listed in Supplementary Information Table S2.

### 2.11. Statistical Analysis

Results are presented as the mean ± standard deviation (SD). Statistical analyses were performed using SPSS 12.0 (SPSS, Inc., Chicago, IL, USA). Differences between groups were assessed by one-way analysis of variance, followed by Duncan’s test. A *p*-value < 0.05 was considered statistically significant.

## 3. Results

### 3.1. LBE and RA Regulate Lipid Metabolism and Oxidative Stress in PA-Treated HepG2 Cells

**Figure 1:**
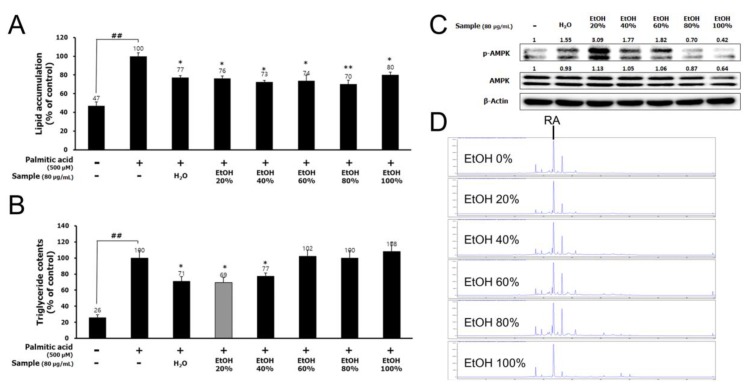
Effects of lemon balm extracts (0%, 20%, 40%, 60%, 80%, or 100% EtOH) on (**A**) lipid accumulation by Oil red-O staining and (**B**) triglyceride (TG) contents in palmitic acid (PA)-treated HepG2 cells. Effects of lemon balm extracts on (**C**) phosphorylation of AMP-activated protein kinase (AMPK) in HepG2 cells. (**D**) The rosmarinic acid (RA) content in the lemon balm extracts by liquid chromatograph. Results are expressed as the mean ± SD of three independent experiments. ^##^ *p* < 0.01 compared with the control group; * *p* < 0.05 and ** *p* < 0.01 compared with the PA-treated cells.

To investigate whether lemon balm extract could suppress lipid accumulation, HepG2 cells were treated with PA alone or with LBEs extracted by 0–100% EtOH (80 μg/mL) for 24 h. Lemon balm extract obtained with 20% EtOH showed the best activity on the suppression of lipid and TG accumulation, and increase of AMPK phosphorylation (Figure 1A–C). Therefore, we utilized the lemon balm extract obtained with 20% EtOH for all subsequent experiments and referred to it as LBE in this paper. However, the content of RA, the major active compound in lemon balm, was not as high in lemon balm extract obtained with 20% EtOH as it was in lemon balm extracts obtained with 40–100% EtOH (Figure 1D and Table 1).

**Figure 2:**
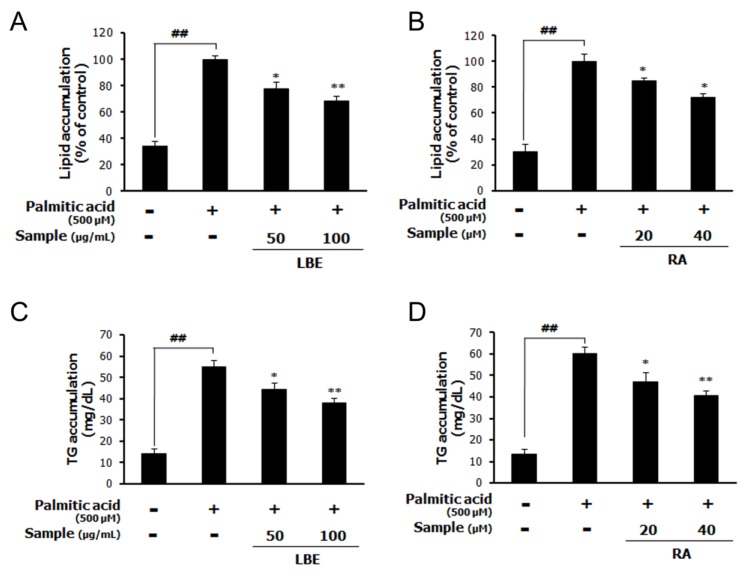
Effects of LBE (lemon balm extract obtained with 20% EtOH) and RA on lipid and TG accumulation in palmitic acid (PA)-treated HepG2 cells. Lipid accumulation with (**A**) LBE and (**B**) RA, and the TG content with (**C**) LBE and (**D**) RA in PA-treated HepG2 cells. Results are expressed as the mean ± SD of three independent experiments. ^##^ *p* < 0.01 compared with the control group; * *p* < 0.05 and ** *p* < 0.01 compared with the PA-treated cells.

The treatment with LBE (the lemon balm extract obtained with 20% EtOH) or RA inhibited the PA-induced upregulation of the accumulation of lipids (Figure 2A,B) and cellular TGs (Figure 2C,D).

**Figure 3:**
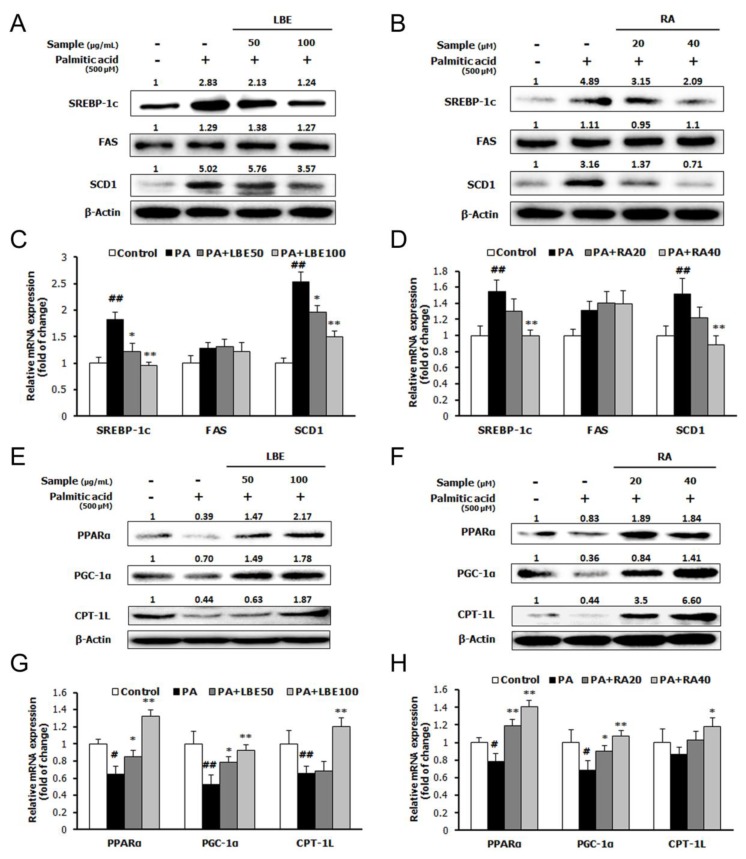
Effects of LBE and RA on the expression of lipid metabolism genes and proteins in HepG2 cells incubated with LBE or RA for 24 h with or without PA. Protein levels of sterol regulatory element-binding protein-1c (SREBP-1c), fatty acid synthase (FAS), and stearoyl-CoA desaturase-1 (SCD-1) with (**A**) LBE and (**B**) RA; peroxisome proliferator-activated receptor α (PPARα), peroxisome proliferator-activated receptor γ coactivator 1α (PGC-1α,) and carnitine palmitoyl transferase 1L (CPT-1L) with (**E**) LBE and (**F**) RA. mRNA levels of the genes encoding *SREBP-1c, FAS,* and *SCD-1* with (**C**) LBE and (**D**) RA*; PPARα, PGC-1α,* and *CPT-1L* with (**G**) LBE and (**H**) RA. β-Actin was used as an internal control for western blotting and qPCR analysis. Results are expressed as the mean ± SD of three independent experiments. ^#^ *p* < 0.05 and ^##^ *p* < 0.01 compared with the control group; * *p* < 0.05 and ** *p* < 0.01 compared with the PA-treated cells.

**Figure 4:**
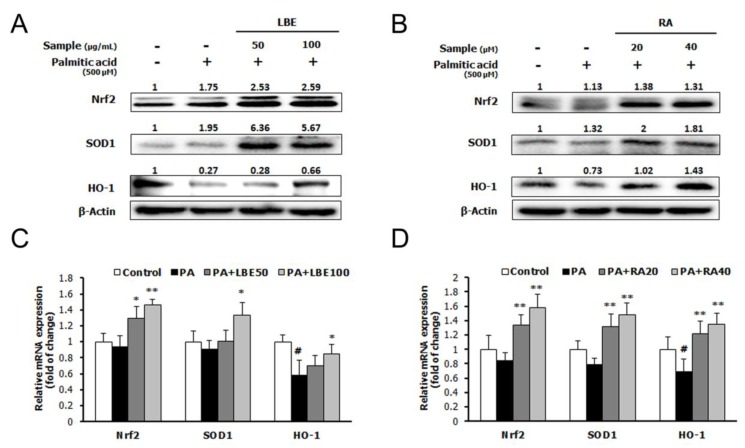
Effects of LBE and RA on the expression of antioxidative stress genes and proteins in HepG2 cells incubated with LBE or RA for 24 h with or without PA. Protein levels of nuclear factor erythroid 2-related factor 2 (NRF2), superoxide dismutase 1 (SOD1), and heme oxygenase 1 (HO-1) with (**A**) LBE and (**B**) RA. mRNA levels of the genes encoding *NRF2, SOD1,* and *HO-1* with (**C**) LBE and (**D**) RA. β-Actin was used as an internal control for western blotting and qPCR analysis. Results are expressed as the mean ± SD of three independent experiments. ^#^ *p* < 0.05 compared with the control group; * *p* < 0.05 and ** *p* < 0.01 compared with the PA-treated cells.

PA treatment increased the expression of lipogenic genes; sterol regulatory element-binding protein-1c (SREBP-1c), fatty acid synthase (FAS), and stearoyl-CoA desaturase-1 (SCD-1) (Figure 3A–D), and suppressed the mRNA and protein expression of lipolytic genes; peroxisome proliferator-activated receptor α (PPARα), peroxisome proliferator-activated receptor γ coactivator 1α (PGC-1α), and carnitine palmitoyl transferase 1L (CPT-1L) (Figure 3E–H). Treatment with LBE or RA reversed these changes in a dose-dependent manner. Moreover, LBE and RA increased the mRNA and protein expression of antioxidant-related genes; NRF2, superoxide dismutase 1 (SOD1), and catalase (Figure 4A–D).

### 3.2. LBE and RA Increase the Level of Phosphorylated AMPK in HepG2 Cells

**Figure 5:**
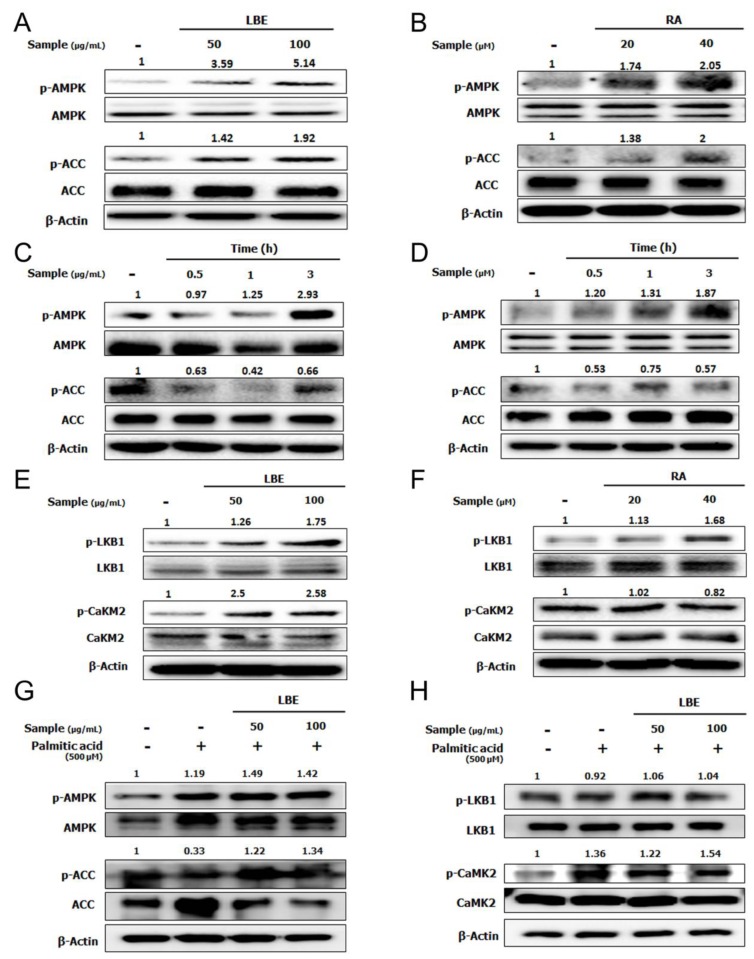
Effects of LBE and RA on AMPK signaling in HepG2 cells without PA. Activation of AMPK and acetyl-CoA carboxylase (ACC) phosphorylation by (**A**) LBE (50 or 100 μg/mL) and (**B**) RA (20 or 40 μM), and (**C**) 100 μg/mL LBE for 0.5–3 h and (**D**) 40 μM RA in for 0.5–3 h. Activation of liver kinase B1 (LKB1) and Ca^2+^/calmodulin-dependent protein kinase II (CaMKII) phosphorylation by (**E**) LBE and (**F**) RA. With PA, **(G)** activation of AMPK and ACC phosphorylation, and **(H)** activation of LKB1 and CaMK2 phosphorylation by LBE (50 or 100 μg/mL). β-Actin was used as an internal control for western blotting.

To elucidate the molecular mechanism by which LBE and RA suppress lipid accumulation, the phosphorylation of AMPK was evaluated in HepG2 cells. Treatment with LBE or RA significantly increased AMPK phosphorylation in a dose- and time-dependent manner (Figure 5A–D). The phosphorylation of acetyl-CoA carboxylase (ACC) was elevated after the treatment with LBE and RA. Among AMPK’s upstream kinases, the levels of liver kinase B1 (LKB1) were increased by both LBE and RA treatments; however, the phosphorylation of Ca^2+^/calmodulin-dependent protein kinase II (CaMKII) was elevated only by LBE (Figure 5E,F). Under PA treatment, LBE also increased the phosphorylated AMPK, the phosphorylated ACC, and the phosphorylated CaMKII, not the phosphorylated LBK1 (Figure 5G,H).

### 3.3. LBE and RA Reduce Liver Damage by Reducing Lipid Accumulation and Hepatic Fibrosis in MCD Diet-Fed db/db Mice

**Figure 6:**
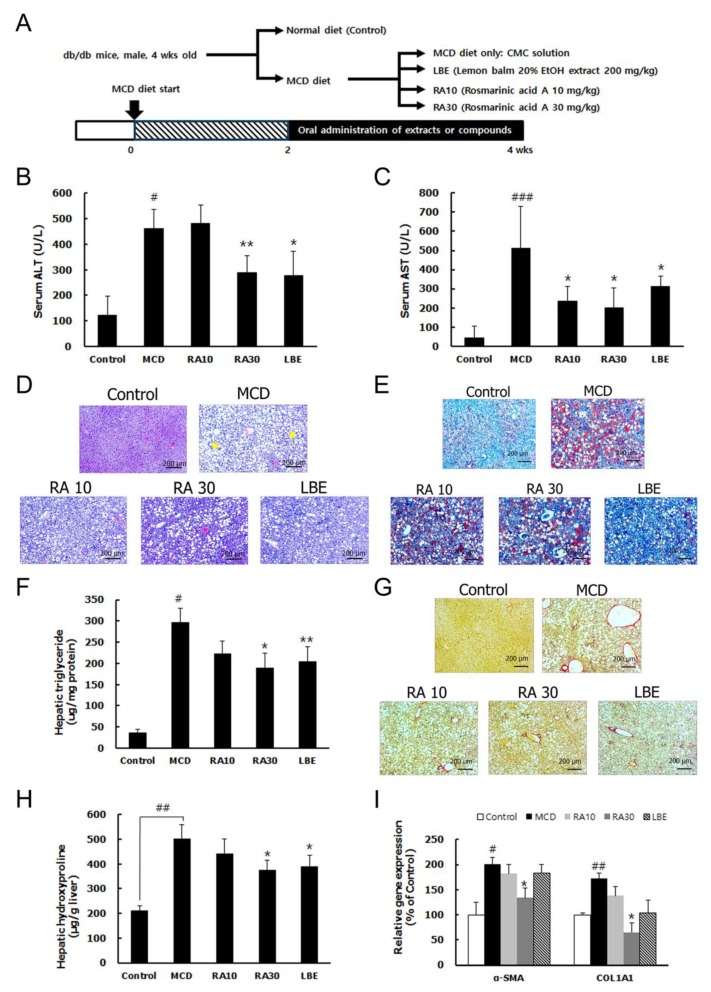
Effect of LBE and RA on liver damage in methionine- and choline- deficient (MCD) diet-fed db/db mice. (**A**) Experimental design of the nonalcoholic steatohepatitis (NASH) animal study. (**B**) Serum alanine transaminase (ALT) and (**C**) aspartate transaminase (AST) levels. (**D**) H&E staining of representative liver sections (arrows indicate inflammatory cells) (magnification: 50×; scale bar: 200 μm). (**E**) Representative images of Oil Red O-stained liver sections (50×; scale bar: 100 μm). (**F**) The liver TG content. (**G**) Collagen deposition, as demonstrated by Sirius Red staining (50×; scale bar: 200 μm). (**H**) The hydroxyproline content in liver sections. (**I**) mRNA expression levels of fibrosis-related genes, normalized to those of β-actin. Results are expressed as the mean ± SD (% control). ^#^ *p* < 0.05, ^##^ *p* < 0.01, and ^###^ *p* < 0.001 compared with the control group; * *p* < 0.05 and ** *p* < 0.01 compared with the MCD diet-fed group (*n* = 5–6 per group).

Consistent with previous reports [20,21], the MCD diet decreased body and liver weights in db/db mice because the MCD diet induced histopathological features of NASH (Figure 6A). The mice fed the MCD diet had significantly elevated serum alanine transaminase (ALT) and aspartate transaminase (AST) levels but LBE and RA30 administration reduced them (Figure 6B,C). H&E and Oil Red O staining showed that hepatic lipid accumulation and the hepatic TG content were significantly higher in the MCD diet-fed mice than in the control mice (Figure 6D–F). Sirius Red staining and an increased level of hepatic hydroxyproline demonstrated the development of hepatic fibrosis. Consistently, hepatic fibrosis markers; α-smooth muscle actin (*α-SMA*) and collagen type I alpha 1 (*COL1A1*), were highly expressed in the mice fed the MCD diet (Figure 6G–I). However, LBE and RA30 suppressed lipid accumulation, the TG content, and fibrosis in the liver of the db/db mice fed the MCD diet (Figure 6D–I). However, RA10 showed no significant effect against liver damage induced by the MCD diet in db/db mice.

### 3.4. LBE and RA30 Reduce NASH via Regulation of the AMPK Pathway, Inflammation, and the NRF2 Pathway in MCD Diet-Fed db/db Mice

**Figure 7:**
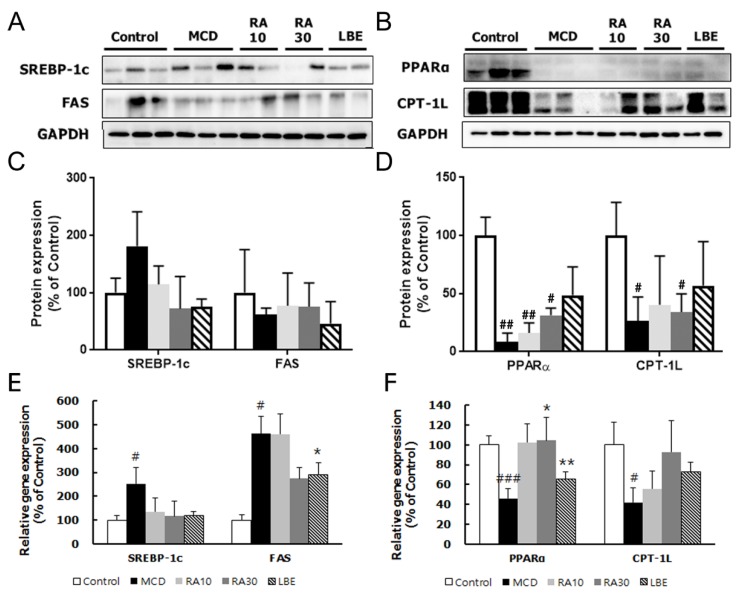
Effects of LBE and RA on the expression of genes and proteins involved in fatty acid regulation in MCD diet-fed db/db mice. Protein levels of (**A**) SREBP-1c and FAS, and (**B**) PPARα and CPT-1L. The densitometric analysis of Western blot of (**C**) SREBP-1c and FAS, and (**D**) PPARα and CPT-1L, normalized to those of glyceraldehyde 3-phosphate dehydrogenase (GAPDH) mRNA levels of the genes encoding (**E**) *SREBP-1c* and *FAS*, and (**F**) *PPARα* and *CPT-1L*. β-Actin was used as an internal control for qPCR analysis. Results are expressed as the mean ± SD (% control). ^#^ *p* < 0.05, ^##^ *p* < 0.01, and ^###^ *p* < 0.001 compared with the control group; * *p* < 0.05 and ** *p* < 0.01 compared with the MCD diet-fed group (*n* = 5–6 per group).

The protein and mRNA expression of lipogenesis genes; SREBP-1c and FAS, was reduced by LBE and RAs (Figure 7A,C). In contrast, the mRNA and protein expression of PPARα and CPT-1L, involved in lipolysis, was increased by LBE and RA (Figure 7B,D). In particular, RA30 escalated the mRNA expression of the *PPARα*, which indicated a stronger impact of RA on lipolysis compared with that of LBE. However, the effects of RA10 on markers of lipid metabolism, except SREBP-1c and PPARα, were not significant.

**Figure 8:**
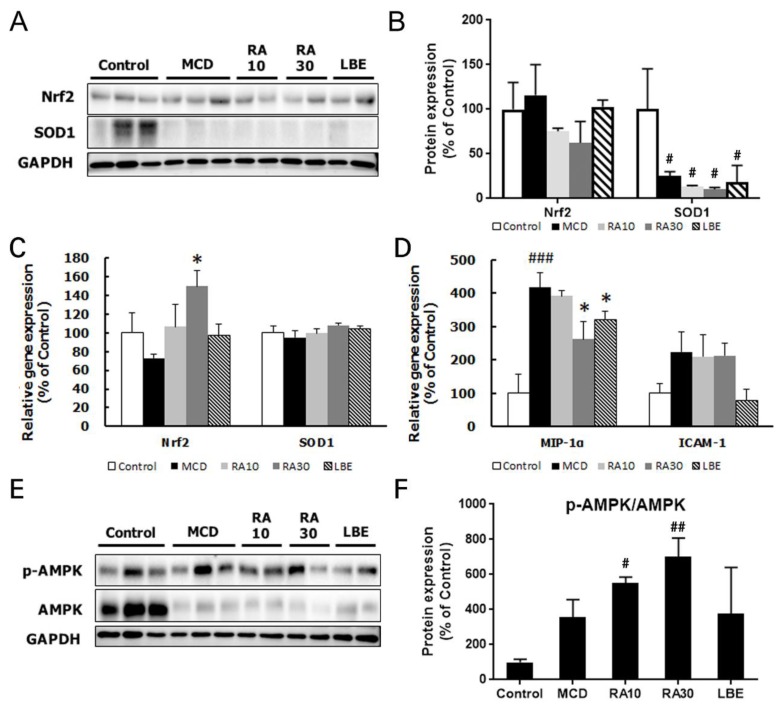
Effects of LBE and RA on the expression of oxidative stress-regulating genes in MCD diet-fed db/db mice. (**A**) Hepatic protein, (**B**) the densitometric analysis of WB, and (**C**) mRNA expression of NRF2 and SOD1. (**D**) Hepatic mRNA expression of macrophage inflammatory protein-1 alpha (*MIP-1ɑ*) and intercellular adhesion molecule 1 (*ICAM-1*). (**E**) Phosphorylation of AMPK and (**F**) the densitometric analysis of WB in MCD diet-fed mice treated with LBE or RA. GAPDH was as an internal control for WB analysis and β-Actin was used for mRNA expression. Results are expressed as the mean ± SD (% control). * *p* < 0.05 compared with the control group; ^#^ *p* < 0.05, ^##^ *p* < 0.01, and ^###^ *p* < 0.001 compared with the control group; * *p* < 0.05 compared with the MCD diet-fed group (*n* = 5–6 per group).

To investigate changes in the antioxidant defense response, we measured the mRNA and protein expression levels of NRF2 and SOD1. The mRNA expression of the NRF2-encoding gene was only increased by RA30, whereas the protein level was elevated by LBE (Figure 8A,B). The gene expression of the inflammatory molecules; macrophage inflammatory protein-1 alpha (*MIP-1α*) and intercellular adhesion molecule 1 (*ICAM-1*), was examined to demonstrate the impacts of LBE and RA on inflammation in the NASH model. The expression of these genes was escalated by the MCD diet but was reduced by LBE and RA30 administration (Figure 8C). The relative phosphorylation of AMPK was enhanced by both LBE and RA (Figure 8D,E). However, the effect of RA30 on the phosphorylation of AMPK was much stronger than that of LBE, indicating that the major role of RA in the NASH model might be a modification of lipid metabolism.

## 4. Discussion

NASH, a complex disease involved in NAFLD, is characterized by excess accumulation of lipids in hepatocytes, along with hepatic inflammation [1]. In the present study, LBE and RA reduced the serum markers of liver damage and suppressed the lipid accumulation as well as fibrosis in the liver, which indicate amelioration of liver damage. These data suggest that LBE and RA can modulate the processes and symptoms associated with NASH.

LBE and RA elevated the levels of LKB1, a kinase phosphorylating AMPK at Thr172. As a result, LBE and RA increased the phosphorylation of AMPK, leading to a decrease in SREBP-1c. Because SREBP-1c is a transcription factor of FAS and SCD1, which are enzymes of fat formation [22], the effects of LBE and RA on the activation of AMPK resulted in the reduction of fat formation via decreases in FAS and SCD1 levels. Furthermore, the activation of AMPK by LBE and RA increased PGC-1α and PPARα, resulting in an increase in CPT-1L [5,23]. Considering that these markers are involved in fatty acid oxidation, the data indicate that LBE and RA may induce fatty acid oxidation. Taken together, LBE and RA suppressed the lipid accumulation in the liver by inhibiting fatty acid synthesis and enhancing fatty acid oxidation, thus leading to the suppression of liver damage.

Excess fatty acids in NAFLD elevate the levels of free fatty acids in hepatocytes, which causes the generation of lipotoxic lipids [3]. Lipotoxic lipids lead to hepatocyte injuries and inflammation, due to increased oxidative stress and stimulation of immune cells in the liver. Therefore, controlling oxidative stress and inflammation is a critical target in the treatment of NASH. NRF2 is a representative detoxifying enzyme, which acts as a transcription factor for antioxidant enzymes, such as SOD1 and heme oxygenase 1 (HO-1) [24]. The increases in NRF2 levels, caused by LBE and RA treatments, indicate their ability to enhance antioxidant mechanisms in the NASH model. Liver injuries by reactive oxygen species (ROS) and lipotoxic lipids result in the immune responses through hepatic macrophages, leading to the activation of hepatic stellate cells [25,26]. These activated hepatic stellate cells recruit circulating immune cells and are differentiated to myofibroblasts, which ultimately results in liver fibrosis.

Suppression of inflammatory molecules, *MIP-1α* and *ICAM-1* by LBE and RA indicates less activated hepatic stellate cells, following less α-SMA and COL1A1, the markers of liver fibrosis. Taken together, LBE and RA can interrupt the progression from fatty liver to NASH through their antioxidant and anti-inflammatory activities. These results confirmed previous studies of RA, in which RA had an anti-fibrotic effect under carbon tetrachloride-induced liver damage and an anti-inflammatory activity on oleic acid-induced NAFLD [27,28].

Although more RA was extracted from lemon balm at higher concentrations of EtOH, we found that the activity of lemon balm extract obtained with 20% EtOH at 200 mg/kg was similar to that of RA at 30 mg/kg in the NASH model, although the concentration of RA in LBE was approximately 4–5% (equivalent to an RA dose of ~10 mg/kg). These data indicate that other small molecules present in LBE contribute to its activity against NASH, even though their content in LBE is very low compared to that of RA. The presence of molecules such as caffeic acid, hesperidin, luteolin, etc., has been reported in LBEs [29]. A number of studies have demonstrated beneficial effects of these compounds; hesperidin and luteolin reduced hepatic steatosis, and epicatechin has antioxidant, anti-inflammatory, and anti-obesity effects [30,31,32,33]. Lithospermic acid, the secondary major compound of LBE has been reported to exhibit an antioxidant activity against oxidative stress [34], but shows no significant effects on lipid accumulation and AMPK activation. Because its concentration was independent of the concentration of water or EtOH, it is unlikely to be a critical factor in the strong effect of LBE against NASH. Therefore, the effect of LBE may be due to synergistic effects of a number of small molecules present in lemon balm, not just one compound.

## 5. Conclusions

We found that LBE and RA exhibited suppressive activities on NASH. They can modulate lipid metabolism via AMPK activation and suppress inflammation via changes in NRF2 signaling. Importantly, the extract of lemon balm obtained with 20% EtOH showed effectiveness similar to that of RA at high concentrations. Therefore, considering the cost of developing and making drugs, LBE may be a good candidate for the treatment and prevention of NASH.

## Figures and Tables

**Table 1:** Difference of RA content between solvents.

Sample	Solvent	g/100 g ext.
**Lemon balm extract**	H_2_O	4.476 ± 0.041
	20% EtOH (LBE)	4.288 ± 0.430
	40% EtOH	4.711 ± 0.006
	60% EtOH	4.865 ± 0.068
	80% EtOH	5.282 ± 0.058
	100% EtOH	5.221 ± 0.016

## Supplementary Materials

The following are available online at https://www.mdpi.com/2072-6643/12/4/1166/s1, Table S1: Antibodies used for western blotting, Table S2: Primers used for real-time qRT-PCR.

## Abbreviations

ACC	acetyl-CoA carboxylase
ALT	alanine aminotransferase
AMPK	AMP-activated protein kinase
AST	aspartate aminotransferase
CAMKII	Ca^2+^/calmodulin-dependent protein kinase II
CoA	coenzyme A
COL1A1	collagen type I alpha 1
CPT-1	carnitine palmitoyl transferase I
CRTC	CREB-regulated transcription coactivator 1
FAS	fatty acid synthase
HO-1	heme oxygenase-1
LBE	lemon balm extract extracted by 20% ethanol
LKB-1	liver kinase B1
MCD diet	methionine- and choline-deficient diet
NAFLD	nonalcoholic fatty liver disease
NASH	nonalcoholic steatohepatitis
NRF2	nuclear factor erythroid-derived 2-related factor 2
PA	palmitic acid
PGC-1	peroxisome proliferator-activated receptor γ coactivator 1
PPARα	peroxisome proliferator-activated receptor α
RA	rosmarinic acid
SCD-1	stearoyl-CoA desaturase-1
α-SMA	α -smooth muscle actin
SOD1	superoxide dismutase 1
SREBP-1	sterol regulatory element-binding protein-1
TG	triglyceride
