# Supplementary material for: Lemon Balm and Its Constituent, Rosmarinic Acid, Alleviate Liver Damage in an Animal Model of Nonalcoholic Steatohepatitis
Source: Nutrients. 2020 Apr 22;12(4):1166. doi: 10.3390/nu12041166 (PMC7230626; doi:10.3390/nu12041166)
Supplement: Supplementary file 1 [file nutrients-12-01166-s001.pdf]

Supplementary Table 1. Antibodies used for western blotting.

Antibody	Company	Catalogue number
SREBP-1c	Santa Cruz Biotechnology	sc-366
FAS	Santa Cruz Biotechnology	sc-715
SCD1	Santa Cruz Biotechnology	sc-14719
PPARα	Santa Cruz Biotechnology	sc-9000
PGC-1α	Santa Cruz Biotechnology	sc-13067
SOD1	Santa Cruz Biotechnology	sc-11407
HO-1	Santa Cruz Biotechnology	sc-1796
GAPDH	Santa Cruz Biotechnology	sc-32233
CPT-1L	Abcam	ab128568
Nrf2	Abcam	ab31163
p-AMPKα (Thr172)	Cell signaling	#2531
AMPKα	Cell signaling	#2532
p-ACC (Ser79)	Cell signaling	#3661
ACC	Cell signaling	#3662
p-LKB1 (Ser428)	Cell signaling	#3482
LKB1	Cell signaling	#3047
p-CaMK2 (Thr286)	Cell signaling	#12716
CaMK2	Cell signaling	#3362

Supplementary Table 2. Primers used for real-time qRT-PCR

Gene	Primer	Sequence
Human Nrf2	Forward	5′-CAG CGA CCT TCG CAA ACA AC-3′
	Reverse	5′-CAT GAT GAG CTG TGG ACC GT-3′
Human SOD1	Forward	5′-ATG GCG ACG AAG GCC GTG TG-3′
	Reverse	5′-GAC CAC CAG TGT GCG GCC AA-3′
Human catalase	Forward	5′-AGC TTA GCG TTC ATC CGT GT-3′
	Reverse	5′-TCC AAT CAT CCG TCA AAA CA-3′
Human SREBP-1c	Forward	5′-ACA TCG AAG GTG AAG TCG GC-3′
	Reverse	5′-GGG AGG GCT TCC TGT AGA GA-3′
Human FAS	Forward	5′-CTC TTG GCC TTT TCC CGG TC-3′
	Reverse	5′-CTC TGA AGC CCA AAG AGG GG-3′
Human SCD-1	Forward	5′-CCC TGC TTA CTT GGT GAG GG-3′
	Reverse	5′-TGC CCT AGG CTG TAG GGA AT-3′
Human PPARα	Forward	5′-AGA AGC TGT CAC CAC AGT AGC-3′
	Reverse	5′-TGA AAG CGT GTC CGT GAT GA-3′
Human PGC-1α	Forward	5′-CTC AGT AAG GGG CTG GTT GC-3′
	Reverse	5′-TCA CTG CAC CAC TTG AGT CC-3′
Human CPT-1L	Forward	5′-CAT ACG AGG CCT CCA TGA CC-3′
	Reverse	5′-CCG TCT CAG GGC AAG AGA AC-3′
Human β-actin	Forward	5′-AGC CAT GTA CGT AGC CAT CC-3′
	Reverse	5′-TCT CAG CTG TGG TGG TGA AG-3′
Mouse SOD1	Forward	5′-TGT GAC TGC TGG AAA GGA CG-3′
	Reverse	5′-ACT GCG CAA TCC CAA TCA CT-3′
Mouse Nrf2	Forward	5′-AAG AAT AAA GTC GCC GCC CA-3′
	Reverse	5′-AGA TAC AAG GTG CTG AGC CG-3′
Mouse PPARα	Forward	5′-CCG AAC ATT GGT GTT CGC AG-3′
	Reverse	5′-AGA TAC GCC CAA ATG CAC CA-3′
Mouse CPT-1L	Forward	5′-GAC TCC GCT CGC TCA TTC C-3′
	Reverse	5′-ACG CCA CTC ACG ATG TTC TT-3′
Mouse SREBP1	Forward	5′-CAG ACT CAC TGC TGC TGA CA-3′
	Reverse	5′-CCT CCA CTC ACC AGG GTC T-3′
Mouse FAS	Forward	5′-CAA GTG TCC ACC AAC AAG CG-3′
	Reverse	5′-GGA GCG CAG GAT AGA CTC AC-3′
Mouse α-SMA	Forward	5′-GTA CCC AGG CAT TGC TGA CA-3′
	Reverse	5′-GAG GCG CTG ATC CAC AAA AC-3′
Mouse COL1A1	Forward	5′-CAC CCC AAT CTG GTT CCC TC-3′
	Reverse	5′-CAT AAG CCA AGT GGG CAG GA-3′
Mouse COL3A1	Forward	5′-GAG GAA TGG GTG GCT ATC CG-3′

	Reverse	5′-TTG CGT CCA TCA AAG CCT CT-3′
Mouse TIMP-1	Forward	5′-CAG ATA CCA TGA TGG CCC CC-3′
	Reverse	5′-CGC TGG TAT AAG GTG GTC TCG-3′
Mouse TGFβ1	Forward	5′-ACT GGA GTT GTA CGG CAG TG-3′
	Reverse	5′-GGA TCC ACT TCC AAC CCA GG-3′
Mouse β-actin	Forward	5′-TAA CCA ACT GGG ACG ATA TG-3′
	Reverse	5′-ATA CAG GGA CAG CAC AGC CT-3′
